# Densest subgraph-based methods for protein-protein interaction hot spot prediction

**DOI:** 10.1186/s12859-022-04996-1

**Published:** 2022-10-31

**Authors:** Ruiming Li, Jung-Yu Lee, Jinn-Moon Yang, Tatsuya Akutsu

**Affiliations:** 1 Bioinformatics Center, Institute for Chemical Research, Kyoto University, Uji, 611-0011 Kyoto Japan; 2 Institute of Bioinformatics and Systems Biology, National Yang Ming Chiao Tung University, Hsinchu, 300 Taiwan; 3 Department of Biological Science and Technology, National Yang Ming Chiao Tung University, Hsinchu, 300 Taiwan

**Keywords:** Bioinformatics, Hot spot, Protein-protein interaction, Residue interaction, Network analysis, Graph theory, Densest subgraph, Linear programming

## Abstract

**Background:**

Hot spots play an important role in protein binding analysis. The residue interaction network is a key point in hot spot prediction, and several graph theory-based methods have been proposed to detect hot spots. Although the existing methods can yield some interesting residues by network analysis, low recall has limited their abilities in finding more potential hot spots.

**Result:**

In this study, we develop three graph theory-based methods to predict hot spots from only a single residue interaction network. We detect the important residues by finding subgraphs with high densities, i.e., high average degrees. Generally, a high degree implies a high binding possibility between protein chains, and thus a subgraph with high density usually relates to binding sites that have a high rate of hot spots. By evaluating the results on 67 complexes from the SKEMPI database, our methods clearly outperform existing graph theory-based methods on recall and F-score. In particular, our main method, Min-SDS, has an average recall of over 0.665 and an f2-score of over 0.364, while the recall and f2-score of the existing methods are less than 0.400 and 0.224, respectively.

**Conclusion:**

The Min-SDS method performs best among all tested methods on the hot spot prediction problem, and all three of our methods provide useful approaches for analyzing bionetworks. In addition, the densest subgraph-based methods predict hot spots with only one residue interaction network, which is constructed from spatial atomic coordinate data to mitigate the shortage of data from wet-lab experiments.

## Background

Proteins realize their functions by interacting with other proteins and/or chemical compounds [1]. Protein-protein interactions play crucial roles in most biological processes. In a protein-protein binding interface, the binding free energy is not uniformly distributed among the residues. Instead, there are hot spots, which contribute most to the binding energy in protein interfaces [2]. Detecting hot spots in protein-protein interactions is meaningful in regulating protein-protein binding and may also contribute to disease control and drug design.

Experimentally, a hot spot residue is defined as having a change in binding energy $\Delta \Delta G \ge 2.0\;{\text{kcal}/\text{mol}}$ upon its mutation to alanine [3]. Several databases have been constructed to collect experimental hot spots from alanine scanning mutagenesis experiments, and two famous databases are the Alanine Scanning Energetics Database (ASEdb) [4] and the Binding Interface Database (BID) [5]. Another widely used database is the SKEMPI database [6], which is new and continually updated (public access to ASEdb and BID is no longer supported). However, finding hot spots by experimental methods is time-consuming and costly; thus, a need for computational methods arises [7].

Several kinds of methods have been designed to predict hot spots. The first type is based on molecular dynamics simulations [8, 9]. Although these methods provide detailed analyses of protein interfaces and have good prediction results, they have difficulty dealing deal with large-scale data because of the high computational cost. Another kind of method is based on energy estimation [10, 11], which estimates the energetic contribution to binding for every interface residue to predict hot spots. Compared to molecular dynamics simulation, energy estimation methods are more efficient in predicting hot spots from large protein complexes.

In recent years, machine learning methods have been frequently used in hot spot prediction, such as extreme gradient boosting [12], random forests [13], and support vector machines (SVMs) [14]. The advantage of machine learning based methods is that they can filter and utilize various possible features to classify residues, together with a well-designed model, and usually have high performance in hot spot prediction. However, since experimentally approved hot spot data are scarce, a large percentage of real hot spot residues are not recognized in hot spot datasets. In machine learning methods, the low rate of positive instances makes it difficult to train models. Additionally, in some methods such as [3, 15–17], to balance the ratio of positive instances to negative ones, only residues with less than $0.4\, \text{kcal}/\text{mol}$ binding free energy are defined as non-hot spots, which further reduces the size of the training set.

On the other hand, there are some methods based on graph theory and network analysis. Tuncbag et al. transformed residue interaction networks into minimum-cut trees and then identified the high-degree nodes as hot spots [18]. Li et al. searched for bicliques from the input network to find highly connected patterns, which have a high possibility of forming a group of hot spots [19]. The graph theory-based methods do not need existing hot spot data to train the models, avoiding the need for many experimental resources, and the prediction results can be a good guide for further biological experiments. Unfortunately, the existing graph theory-based methods have very low recall. Although some hot spots can be precisely detected by these methods, many possible hot spots are ignored.

Here, we consider using other graph theory methods, which are based on the densities of subgraphs, to analyze residue interaction networks. Generally, high density refers to a high connectivity between vertices, and it often relates to binding sites in complexes. By further evaluation, we find that our methods have an obvious advantage in finding potential hot spots, as well as having similar precision to that of the existing methods. The results of these densest subgraph-based methods (DS-based methods) can be a good reference for future bio-experiments.

Generally, a certain input network may contain multiple densest subgraphs. We can simply select one random densest subgraph as an output. In this research, we use DS to represent this random method. However, because of this randomness, it is difficult to ensure the performance of the DS. To obtain better performance in practice, we propose three variant methods based on the DS method (DS-based methods). The first method yields all the minimal densest subgraphs [20] as the result, and we use Min-DS to denote this method. Compared to DS, Min-DS has no randomness and has better precision and recall than DS. The second method, Max-DS, is based on a novel concept, namely, the maximal densest subgraph. The results of Max-DS include those of Min-DS, and it has higher recall but lower precision than Min-DS. To maximize the ability to find potential hot spots, we develop a third method, Min-SDS, which is also the main method in our research. This method is similar to Min-DS but has a weakened restriction in detecting the minimal densest subgraph. By further evaluation, we find that Min-SDS has the best recall and F2-score among all the graph theory-based methods and performs well in detecting unknown hot spots.

## Result

### Dataset

We mainly use the data from the SKEMPI 2.0 dataset [6], which records 7085 pieces of mutation information on 341 protein complexes, to define the hot spots in protein complexes. Specifically, if a residue has $\Delta \Delta G=\Delta G_{mut}-\Delta G_{wt} \ge 2.0 \,\text{kcal}/\text{mol}$ in an alanine-mutation experiment, then this residue is recognized as a hot spot [4]. Here, $\Delta G_{wt}$ and $\Delta G_{mut}$ are the binding free energies upon complex formation of the wild-type and alanine-mutated proteins, respectively. $\Delta G$ can be calculated by $\Delta G=RT \ln {Kd}$, where *R* is the ideal gas constant, *T* is the absolute temperature, and *Kd* is the affinity of the wild-type (*wt*) or mutant (*mut*) complexes. Thus, we have $\Delta G_{wt}=(8.314/4184)*(273.15+25.0)*\ln (wt), \Delta G_{mut}=(8.314/4184)*(273.15+25.0)*\ln (mut)$ [21].

The residue interaction network data are based on PDB spatial data [22]. In a protein complex, any two residues in different chains are regarded as contacting each other if there exist two atoms *a* and *b* from each residue such that their distance $d(a,b) \le r(a)+r(b)+2.75 {\text{\AA} }$, where *r* is the van der Waals radius, and $2.75 {\text{\AA} }$ is the diameter of a water molecule [19]. To build a residue interaction network for each protein complex, only the residues that contact at least one other residue are selected as vertices of the network, and an edge is added between any two contacting vertices.

The atom spatial data in PDB are based on crystal artifacts, sometimes they may not directly reflect the natural protein quaternary structure of complexes [23, 24]. To avoid the problem of choosing proper biological assemblies among asymmetric units, we selected 223 complexes from the 341 complexes, each of which has only one possible biological assembly, to construct residue interaction networks. We further selected 67 networks with at least 3 bio-experimentally approved hot spots for evaluation.

In addition, we used another independent hot spot dataset, AB-bind [25], for evaluation, which contains 1101 mutation records on 27 complexes. Using the same data selection strategy, 5 complexes were selected for result evaluation.

### Experiments and evaluation

We implemented the DS, Min-DS, Max-DS, Min-SDS, Biclique, and Mincut methods on the built networks.

The DS method finds a random densest subgraph of the input network; the Min-DS method finds all the minimal densest subgraphs [20]; the Max-DS method finds the maximal densest subgraph; and the Min-SDS method finds a set of nonintersecting subgraphs with high densities.

Biclique and Mincut are existing methods. The Biclique method [19] finds all the bicliques of the input network. In our experiments, only the bicliques that contain at least 3 vertices on each side are selected as the result. The Mincut method [18] first builds the mincut tree of the input network, and then the high-degree (at least degree 3) nodes in the tree are selected as the result.

**Fig. 1:**
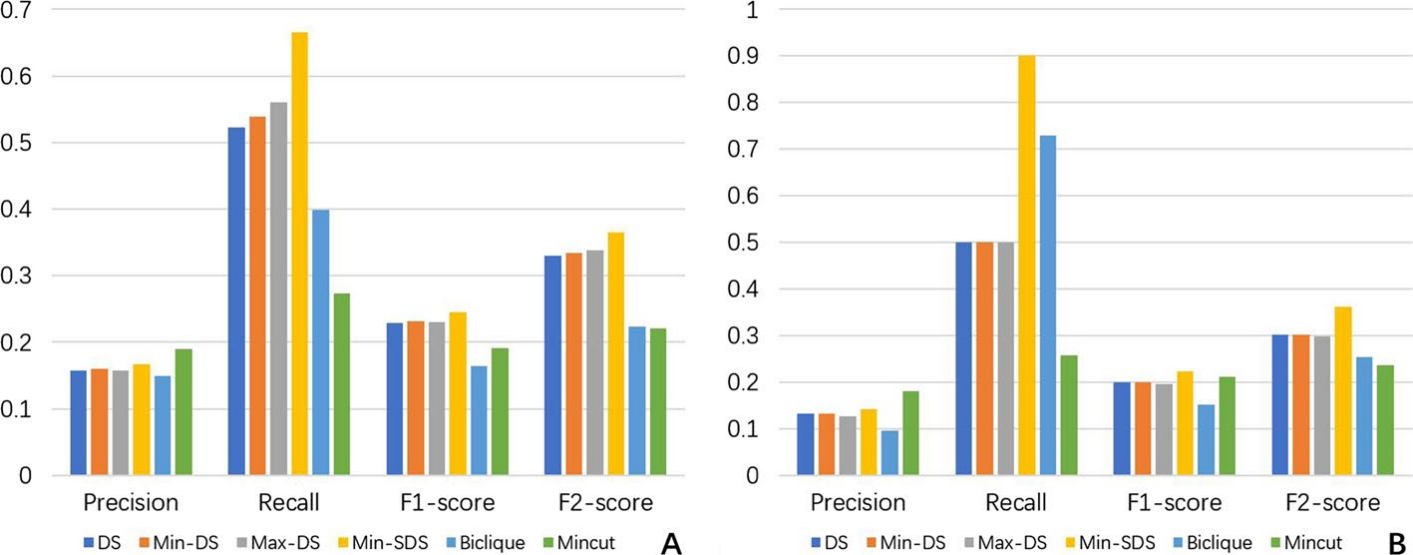
Clustered column chart of the performances of each method on SKEMPI (**A**) and AB-bind (**B**). The result distributions on the two charts are similar. In both datasets, Min-SDS has the best recall and F-score, and all DS-based methods outperform the existing methods in terms of F2-score

The average results of all six methods are shown in Fig. 1 ($\theta =0.85$ for Min-SDS). A detailed definition of each standard metric can be found in Additional file 1. The data of the results of all the methods can be found in Additional files 2, 3, 4, 5.

Compared to the existing methods, our DS-based methods have much better F-scores. Although Mincut has the best precision, its recall is very low compared to the other methods. In hot spot research, there is a lack of bio-experiments detecting whether a residue is a hot spot. Even if some experiments on a residue have been performed and indicated $\Delta \Delta G < 2.0 \text{kcal}/\text{mol}$, it is difficult to determine that this residue is not a hot spot. Many potential hot spots may be false-negatively tagged by bio-experiments. In this situation, higher recall should be more beneficial than higher precision.

Another disadvantage of Mincut is that its results tend to be in one connected component. However, a protein complex may have multiple binding sites, which means that several distinct subgraphs may contain hot spots, while in most cases, the Mincut method focuses on only one of them.

**Fig. 2:**
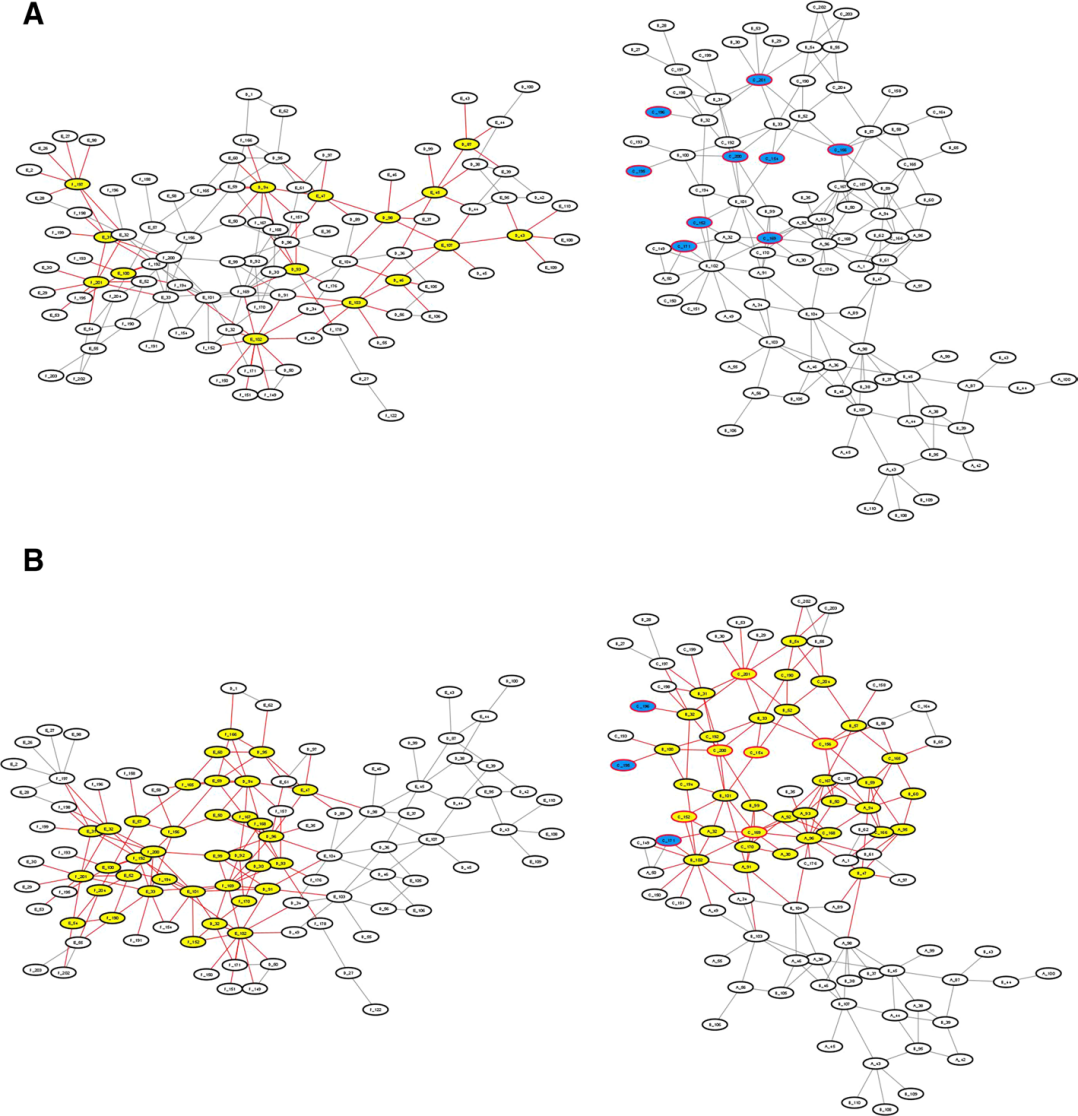
The results of Mincut and Min-SDS on the graph of complex 1AHW. TP: red outline, yellow fill; FP: black outline, yellow fill; TN: black outline, white-fill; FN: red outline, blue fill. These figures show only part of the 1AHW network

As an example, complex 1AHW [26] consists of 3 molecules, and each molecule has 2 chains (AD, BE and CF). These 6 chains compose a heterohexamer (preferred) biological assembly composition. By checking the residue interaction network, 5 large connected subgraphs are found to exist: A-B, D-E, A-F, A-B-C and D-E-F (subgraph A-B means that all the residues in this subgraph come from chain A or B, and the other terms have similar meanings). In these subgraphs, A-B-C and D-E-F are highly connected, and both of them have high possibilities of containing hot spots. In fact, all the experimentally approved hot spots are gathered in the A-B-C subgraph. However, in practice, the Mincut method only predicts residues in the D-E-F area and thus performs poorly in this instance. For details, see Fig. 2.

Our DS-based methods, especially the Min-SDS method, are not restricted to only one connected area, and thus all highly connected areas can be selected. For instance 1AHW, the result of the Min-SDS method distributes in both A-B-C and D-E-F subgraphs, successfully covers the approved hot spots, and predicts the possible hot spots in the D-E-F area.

Since the Min-SDS method removes the restriction of ‘densest’, it has the best advantage in finding possible hot spots. In our experiments, the tolerance $\theta$ of Min-SDS was set to 0.85; i.e., all minimal subgraphs with a density higher than $0.85*D$ were selected, where *D* is the maximum density of the input graph.

**Fig. 3:**
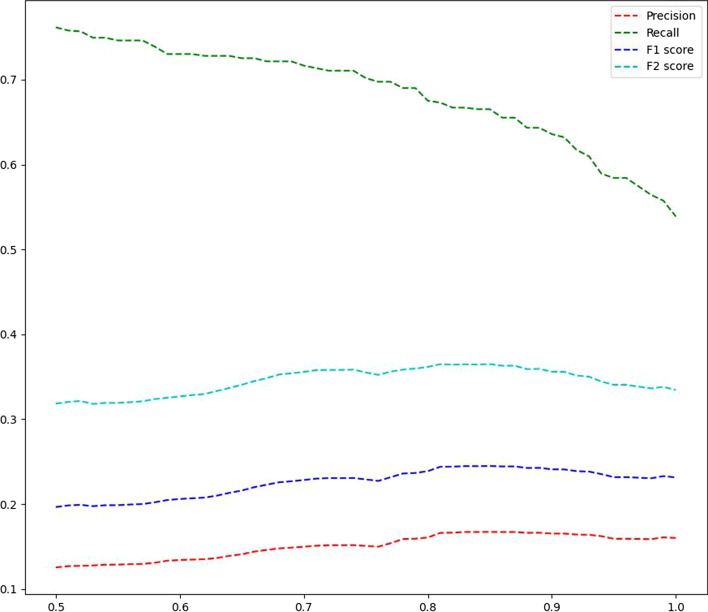
The average performances of Min-SDS on different $\theta$ values (x-axis). The F2-score peaks at $\theta =0.85$

We tested the performance of Min-SDS on different $\theta$ values from 0.5 to 1.0, and the results are shown in Fig. 3. With the decrease in $\theta$, the precision decreases while the recall increases. The F2-score peaks when $\theta = 0.85$; this score is the best among those of all DS-based methods, and is obviously better than those of the existing methods.

**Fig. 4:**
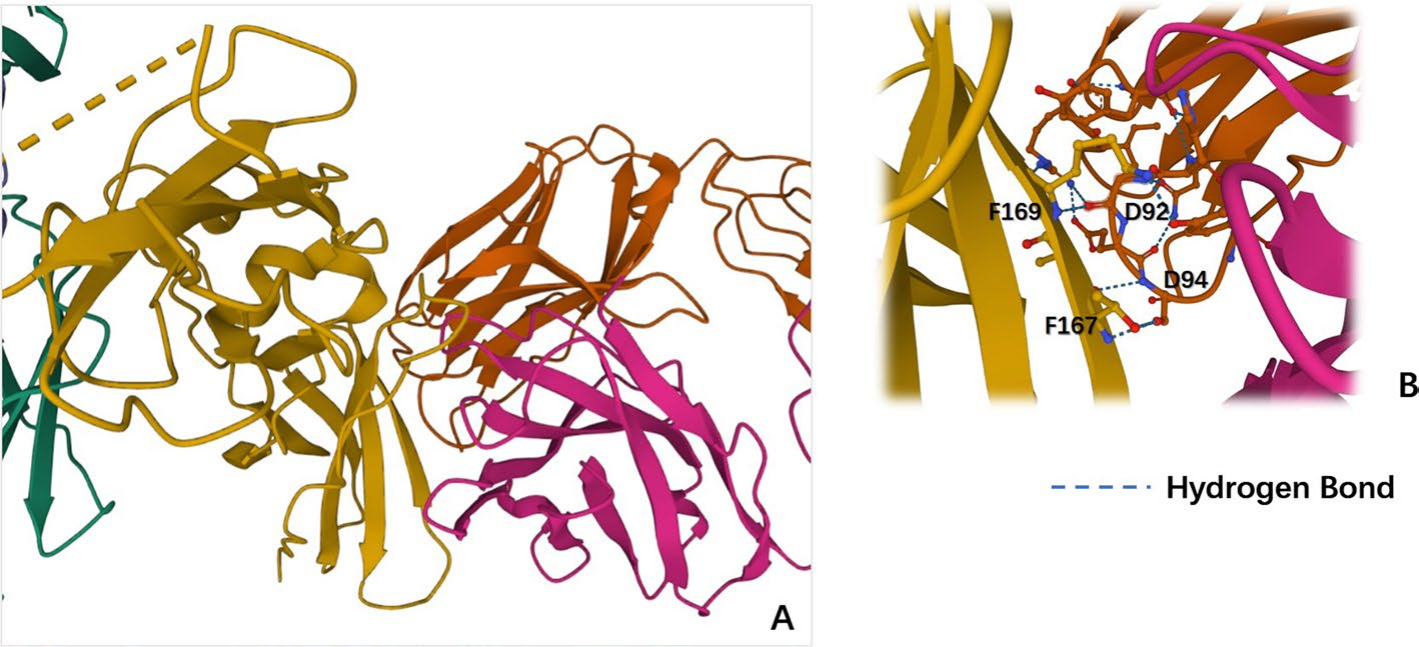
A 3D view of the D-E-F area of 1AHW **A** A 3D view of the quaternary structure of 1AHW in the D-E-F area;** B** Hydrogen bonds between chains D and F

By further analyzing the 3D view of the protein complexes, we can see that the Min-SDS method does have the advantage of predicting unknown hot spots. In the same instance, 1AHW, the Min-SDS method predicts 36 residues in the D-E-F area. Of these residues, 18 form hydrogen bonds with residues from another chain (Fig. 4). To estimate whether a residue is a hot spot, the change in the binding energy from residue mutation is the only metric used. The energy of a hydrogen bond varies from $\approx 5\sim 6\, \text{kcal}/\text{mol}$ for the isolated bond to $\approx 0.5\sim 1.5\, \text{kcal}/\text{mol}$ for proteins in solution [27], close to the threshold $2.0\, \text{kcal}/\text{mol}$. When a residue forms a hydrogen bond to another chain, the mutation of this residue will obviously influence the generation of the wild-type hydrogen bond, which should significantly change the binding energy between the chains. Thus, many of the predicted residues in the D-E-F area have the potential to be hot spots.

## Conclusion

In this study, we develop three densest subgraph-based methods for protein-protein interaction hot spot prediction. Compared to the existing graph theory-based methods, our methods perform much better in terms of recall and F-score. In particular, our Min-SDS method has an obvious advantage in terms of recall and has the best F2-score among all the graph theory-based methods. In addition, our Min-DS and Max-DS methods outperform the existing methods in terms of F-score, providing useful network analysis methods for researchers.

Although the Mincut method has the best precision, its predictions tend to be concentrated in one connected subgraph, which significantly reduces the recall in practice. In comparison, the results of our DS-based methods are not restricted to one connected component, which is important in dealing with complexes with multiple binding sites.

Compared to machine learning methods, our DS-based methods do not depend on insufficient bio-experimental data and thus have the advantage of being able to search unknown hot spots without many data resources.

Our DS-based methods use only spatial coordinate information to detect important vertices in a given interaction network. The high recall scores make them good choices for some other high-false-negative-rate networks analyses, and they can be easily applied to various network analysis fields.

## Method

### Problem transformation

For a given protein complex, we first convert the residue spatial coordinate information to an undirected graph, where the vertices correspond to the residues and the edges correspond to the contacts between residues. Then, the hot spot prediction problem is transformed into the problem of searching for critical vertices in an input graph, and the selected vertices correspond to the predicted hot spot residues.

### Densest subgraph

Given an undirected graph $G=(V,E)$, where $V=\{ 1,2,...,n \}$ is the set of vertices and *E* is the set of edges of *G*, the density of *G* is defined as $\rho (G)=\frac{|E|}{|V|}$. Let *S* be a subgraph of *G*. If *S* has the maximum density among all possible subgraphs of *G*, then *S* is a densest subgraph of *G*. A certain graph *G* may have multiple densest subgraphs.

In [28], a linear programming (LP)-based method was proposed to search for a densest subgraph of *G*. For each edge $(i,j) \in E$, a real-valued variable $x_{i,j} \in [0,1]$ is set, and for each vertex $i \in V$, a real-valued variable $y_i \in [0,1]$ is set. Then, the following LP method returns a solution that contains the information of a random densest subgraph of *G*: **BasicLP**(*V*, *E*)

$$\begin{aligned} {\textbf {Maximize}}&\sum \nolimits_{(i,j) \in E} x_{i,j}&\\ {\textbf {Subject to}}& \, x_{i,j} \le y_i&\forall (i,j) \in E \\& \, x_{i,j} \le y_j&\forall (i,j) \in E \\&\sum \nolimits_{i \in V} y_i \le 1&\\&x_{i,j} \ge 0, y_i \ge 0&\forall i,j \end{aligned}$$

For an optimal solution of BasicLP, the set of vertices $S=\{i \in V|y_i>0 \}$induces a densest subgraph of *G*. We also use DS to denote this LP-based method.

Furthermore, we have the following proposition:

#### Proposition 1

For any optimal solution of *BasicLP*, the set of vertices $S=\{i \in V|y_i \ge \frac{1}{|V|} \}$ induces a densest subgraph of G.

Accordingly, in practice, we select the vertices with $y_i \ge \frac{1}{|V|}$ rather than $y_i>0$ as the output because of the numerical error of the Gurobi solver [29].

The proof of Proposition 1 can be found in Additional file 1.

### Minimal densest subgraph

Given an undirected graph $G=(V,E)$, let *S* be a densest subgraph of *G*. If for any subgraph $S'$ of *S*, $\rho (S') < \rho (S)$, then *S* is a minimal densest subgraph. One graph may include multiple minimal densest subgraphs. In [20], Balalau et al. presented an LP-based method to find all minimal densest subgraphs for an input graph. We use Min-DS to denote this method.

### Maximal densest subgraph

Given an undirected graph $G=(V,E)$, let *S* be a densest subgraph of *G*. If any densest subgraph of *G* is a subgraph of *S*, then *S* is the maximal densest subgraph.

#### Proposition 2

For any undirected graph, exactly one maximal densest subgraph exists.

The proof of Proposition 2 can be found in Additional file 1.

We can find the maximal densest subgraph of an input graph $G=(V,E)$ by an integer linear programming (ILP)-based method. For each edge $(i,j)\in E$, we set a real-valued variable $x_{i,j} \in [0,1]$; for each vertex $i \in V$, we set a real-valued variable $y_i \in [0,1]$ and a Boolean variable $z_i$. Let *D* be the maximum density of *G* (we can obtain *D* by BasicLP). Then, we have the following ILP:

**MaxILP**

1 $$\begin{aligned} {\textbf {Maximize}}&\sum\nolimits _{i \in V} z_i&\nonumber \\ {\textbf {Subject to}}& \, x_{i,j} \le y_i&\forall (i,j) \in E \end{aligned}$$

2 $$\begin{aligned}&x_{i,j} \le y_j&\forall (i,j) \in E \end{aligned}$$

3 $$\begin{aligned}&\sum\nolimits _{i \in V} y_i \le 1&\end{aligned}$$

4 $$\begin{aligned}&x_{i,j} \ge 0, y_i \ge 0&\forall i,j \end{aligned}$$

5 $$\begin{aligned}&\sum\nolimits _{(i,j) \in E} x_{i,j} \ge D&\end{aligned}$$

6 $$\begin{aligned}&y_i - \frac{z_i}{|V|} \ge 0&\forall i \in V \end{aligned}$$

This ILP method is denoted as Max-DS. Furthermore, we have proposition 3.

#### Proposition 3

For an optimal solution $H=(x^H,y^H,z^H)$ of *MaxILP*, the set of vertices $\{i|z_i \in z^H,z_i=1\}$ induces the maximal densest subgraph of *G*.

The proof of Proposition 3 can be found in Additional file 1.

We can also use an LP-based method to find the maximal densest subgraph. First, we modify BasicLP to the following LP (the definition of the variables is the same as in BasicLP):

**MaxLP** (*V*, *E*, *D*, *R*)

7 $$\begin{aligned} {\textbf {Maximize}}&\sum\nolimits _{(i,j) \in E} x_{i,j}&\nonumber \\ {\textbf {Subject to}}& \, x_{i,j} \le y_i&\forall (i,j) \in E \end{aligned}$$

8 $$\begin{aligned}&x_{i,j} \le y_j&\forall (i,j) \in E \end{aligned}$$

9 $$\begin{aligned}&\sum\nolimits _{i \in V} y_i \le 1&\end{aligned}$$

10 $$\begin{aligned}&x_{i,j} \ge 0, y_i \ge 0&\forall i,j \end{aligned}$$

11 $$\begin{aligned}&\sum\nolimits _{(i,j) \in E} x_{i,j} \ge D&\end{aligned}$$

12 $$\begin{aligned}&y_i \ge \frac{1}{|V|}&i \in R \end{aligned}$$

13 $$\begin{aligned}&\sum\nolimits _{i \in V-R} y_i \ge \frac{1}{|V|}&\end{aligned}$$

Here, *D* is the density of the input graph, and *R* is a subset of *V*. Compared to BasicLP, we add constraints (11)–(13) to the program. Constraint (11) requires that the solution leads to a densest subgraph; constraint (12) requires that all the vertices in *R* should be selected to the solution; constraint (13) requires that at least one vertex that is not in *R* should be selected.

We set the objective value as the return of BasicLP and use $\{ i|i \in V,z_i=1 \}$ or $\emptyset$ (if no feasible solution is found) as the return of MaxLP.

Then, we have the algorithm FindMaximal.
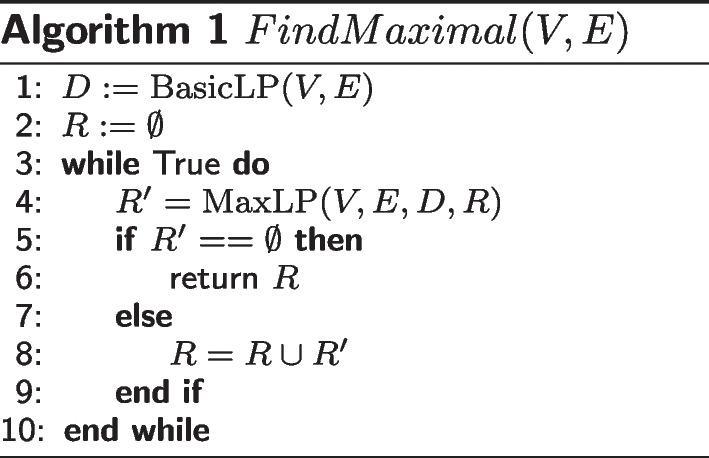


The correctness of FindMaximal is obvious. In the worst case, we need to run MaxLP *O*(*n*) times, and thus we can solve the problem in polynomial time.

In practice, the MaxILP and FindMaximal methods have very similar time costs, and thus the evaluation is based on the results of MaxILP, which is easier to implement (although both methods have the same results because of the uniqueness of the maximal densest subgraph).

### Minimal sub-densest subgraph

In some protein complexes, multiple binding interfaces may exist, while in the residue interaction network, the interface areas may have different densities. If we always search the densest subgraph, some hot spots in some binding interfaces may be ignored.

Here, we consider weakening the restrictions of in Min-DS to find more potential hot spots. The skeleton of Min-DS is as follows [20]:

1. $$result:= \emptyset$$.
2. Find a minimal densest subgraph *R*.
3. If $\rho (R) < \rho (G)$, then return *result*; otherwise, set $result = result \cup R,$ remove *R* from the graph, and then jump to step 2.

In step 3, if Min-DS has a smaller density than the input graph, then the process stops. Here, we consider adding a tolerance $\theta$ to step 3 as follows:

1. $$result:= \emptyset$$.
2. Find a minimal densest subgraph *R*.
3. If $\rho (R) < \theta * \rho (G)$, where $0< \theta < 1$, then return *result*; otherwise, set $result = result \cup R,$ remove *R* from the graph, and then jump to step 2.

We call the result the minimal sub-densest subgraphs, and this method is named Min-SDS.

### Experimental environment

We implemented all the methods in Python 3.10 with an Intel(R) Core(TM) i5-10210U CPU and 8.00 GB RAM. The LP and ILP processes are based on Gurobi 9 [29].

## Supplementary Information

**Additional file 1**. Proofs of propositions 1 to 3; Definitions of standard metrics.

**Additional file 2**. Recalls of all the methods on all the complexes.

**Additional file 3**. Precisions of all the methods on all the complexes.

**Additional file 4**. F1-scores of all the methods on all the complexes.

**Additional file 5**. F2-scores of all the methods on all the complexes.

## Data Availability

The code and experimental data are available on Github: https://github.com/lrming1993/DensestSubgraphCode The details of the recall, precision, F1-score and F2-score of each method on all complexes can be found in the additional files.

## Abbreviations

DS: Densest subgraph method
DS-based: Densest subgraph-based method
Min-DS: Minimal densest subgraph method
Max-DS: Maximal densest subgraph method
Min-SDS: Minimal sub-densest subgraph method
LP: Linear programming
ILP: Integer linear programming
