# Supplementary material for: Densest subgraph-based methods for protein-protein interaction hot spot prediction
Source: BMC Bioinformatics. 2022 Oct 31;23:451. doi: 10.1186/s12859-022-04996-1 (PMC9623998; doi:10.1186/s12859-022-04996-1)
Supplement: Supplementary file 1 — Additional file 1. Proofs of propositions 1 to 3; Definitions of standard metrics. [file 12859_2022_4996_MOESM1_ESM.docx]

**Proposition 1** For any optimal solution of BasicLP, the set of vertices $S=\{i\in V|y_{i}\geq\frac{1}{\left| V \right|}\}$ induces a densest subgraph of $G$.

**Proof.** For any optimal solution, obviously, we have $\sum_{i\in V} y_{i}=1$, thus if there exists some $y_{i}<\frac{1}{\left| V \right|}$, there must exist some $y_{j}>\frac{1}{|V|}$.

According to the proof of Lemma 4.1 of [2], when an optimal solution has different non-zero values of $y_{i}$’s, if we remove the $y_{i}$’s with the lowest non-zero value (set them to 0), the remaining $y_{i}$’s with non-zero value(s) still correspond to a densest subgraph. We can repeat the process until the lowest non-zero value is larger than $\frac{1}{\left| V \right|}$, and the remaining $y_{i}$’s with non-zero values correspond to a densest subgraph. $∎$

**Proposition 2** For any undirected graph, exactly one maximal densest subgraph exists.

**Proof.** For a given graph $G$, the union of all possible densest subgraphs is obviously a maximal densest subgraph. However, there may exist another maximal densest subgraph.

Assume more than one maximal densest subgraph exists. Let $S_{1}$ and $S_{2}$ be two different maximal densest subgraphs. According to Corollary 4.1 of [2], $S_{3}:=S_{1}\cup S_{2}$ is also a densest subgraph. Since $S_{1}\neq S_{2}$, $S_{3}$ is not a subgraph of $S_{1}$ or $S_{2}$, thus $S_{1}$ and $S_{2}$ are not maximal densest subgraphs, which contradicts to the assumption. Therefore, only one maximal densest subgraph exists. $∎$

**Proposition 3** For an optimal solution $H=(x^{H},y^{H},z^{H})$ of MaxILP, the set of vertices $\{i|z_{i}\in z^{H},z_{i}=1\}$ induces the maximal densest subgraph of $G$.

**Proof.** Let $S$ be the maximal densest subgraph, then the following solution $(x^{S},y^{S},z^{S})$ is a feasible solution of MaxILP:

$x_{i,j}^{S}=\left\{ \begin{matrix} \frac{1}{|S|} & if both i\in S \mathrm{and}j\in S \\ 0 & \mathrm{otherwise} \end{matrix} \right.$

$y_{i}^{S}=\left\{ \begin{matrix} \frac{1}{|S|} & \mathrm{if}i\in S \\ 0 & \mathrm{otherwise} \end{matrix} \right.$

$z_{i}^{S}=\left\{ \begin{matrix} 1 & \mathrm{if}i\in S \\ 0 & \mathrm{otherwise} \end{matrix} \right.$

Constraints (1), (2) and (4) restrict the subgraph induced by $\{i|i\in V,y_{i}\geq\frac{1}{|V|}\}$ be a densest subgraph (by **Proposition 1**). By constraint (3), $z_{i}=1$ only if $y_{i}\geq\frac{1}{|V|}$. Thus $\sum_{i\in V} z_{i}\leq|S|$, otherwise, the set $\{i|i\in V,y_{i}\geq\frac{1}{|V|}\}$ induces a densest subgraph that has a size larger than $|S|$, which is impossible. $∎$

**Definition of standard metrics:**

Let $TP$, $TN$, $FP$ and $FN$ be the numbers of true positive, true negative, false positive and false negative residues in the predictions, respectively. The standard metrics are defined as follows:

$$\mathrm{Precision}=\frac{TP}{TP+FP}$$

$$\mathrm{Recall}=\frac{TP}{TP+FN}$$

$$\text{F1-Score}=\frac{2\times Precision\times\mathrm{Recall}}{\mathrm{Precision}+\mathrm{Recall}}$$

$$\text{F2-Score}=\frac{5\times Precision\times Recall}{4\times Precision+Recall}$$
